# COMPENSATION EFFECT OF MORTALITY: A CHALLENGE TO LIFE EXTENSION

**DOI:** 10.1093/geroni/igz038.3504

**Published:** 2019-11-08

**Authors:** Natalia S Gavrilova, Leonid Gavrilov

**Affiliations:** NORC at the University of Chicago, Chicago, Illinois, United States

## Abstract

In order to develop genuine anti-aging interventions it is important to find the best estimate of the aging rate in humans, which is often measured as a slope parameter of the Gompertz law. The compensation effect of mortality (CEM), refers to mortality convergence, when higher values for the slope parameter are compensated by lower values of the intercept parameter (initial mortality) in different populations of a given species. The age of this convergence point is called the "species-specific life span". Due to CEM, factors associated with life span extension are usually accompanied by paradoxical increase in actuarial aging rate. We evaluated the stability of CEM by analyzing the United Nations abridged life tables for 241 countries and regions and estimating parameters of the Gompertz-Makeham model using method of non-linear regression in the age interval 30-80 years. We found that the species-specific lifespan is equal to 94.5 ± 0.5 years, which is the same as reported in the past for years before the 1960s: 95 ± 3 years (Gavrilov, Gavrilova, 1991). Thus, the convergence point of CEM is stable despite significant mortality decline over past 50 years and is not affected by factors decreasing mortality at younger ages. Populations deviating from CEM with apparently slow aging (with both slow actuarial aging rate and low intercept parameter) have been identified. The existence of CEM in mice (ITP data) allowed us to find interventions that are able to both extend lifespan and slow the actuarial aging rate giving promise for radical life extension.

